# Evolution of the Targeted Therapy Landscape for Cholangiocarcinoma: Is Cholangiocarcinoma the ‘NSCLC’ of GI Oncology?

**DOI:** 10.3390/cancers15051578

**Published:** 2023-03-03

**Authors:** Amol Gupta, Razelle Kurzrock, Jacob J. Adashek

**Affiliations:** 1 Department of Medicine, The Sidney Kimmel Comprehensive Cancer Center, The Johns Hopkins Hospital, Baltimore, MD 21287, USA; 2 WIN Consortium, San Diego, CA 92093, USA; 3 Division of Hematology and Oncology, Medical College of Wisconsin Cancer Center, Milwaukee, WI 53226, USA; 4 Division of Hematology and Oncology, University of Nebraska, Omaha, NE 68182, USA; 5 Department of Oncology, The Sidney Kimmel Comprehensive Cancer Center, The Johns Hopkins Hospital, Baltimore, MD 21287, USA

**Keywords:** cholangiocarcinoma, targeted therapy, molecular biomarkers

## Abstract

**Simple Summary:**

In the past 20 years, the development of targeted therapies that can be matched to a tumor’s molecular and immune abnormalities has resulted in the improvement of outcomes for patients suffering from advanced aggressive malignancies. Remarkably, non-small cell lung cancer (NSCLC) has become the poster child for a lethal malignancy in which numerous molecular aberrations have become druggable. Similar to NSCLC, there are limited responses in cholangiocarcinoma (CCA) to conventional chemotherapy. Next-generation sequencing has identified novel genomic alterations in CCA that vary between patients. Gene- and immune-targeted therapies are leading to a new era of precision/personalized medicine for patients with CCA. Herein, we review the current status of molecularly matched precision-targeted therapy for CCA.

**Abstract:**

In the past two decades, molecular targeted therapy has revolutionized the treatment landscape of several malignancies. Lethal malignancies such as non-small cell lung cancer (NSCLC) have become a model for precision-matched immune- and gene-targeted therapies. Multiple small subgroups of NSCLC defined by their genomic aberrations are now recognized; remarkably, taken together, almost 70% of NSCLCs now have a druggable anomaly. Cholangiocarcinoma (CCA) is a rare tumor with a poor prognosis. Novel molecular alterations have been recently identified in patients with CCA, and the potential for targeted therapy is being realized. In 2019, a fibroblast growth factor receptor 2 (*FGFR2*) inhibitor, pemigatinib, was the first approved targeted therapy for patients with locally advanced or metastatic intrahepatic CCA who had *FGFR2* gene fusions or rearrangement. More regulatory approvals for matched targeted therapies as second-line or subsequent treatments in advanced CCA followed, including additional drugs that target *FGFR2* gene fusion/rearrangement. Recent tumor-agnostic approvals include (but are not limited to) drugs that target mutations/rearrangements in the following genes and are hence applicable to CCA: isocitrate dehydrogenase 1 (*IDH1*); neurotrophic tropomyosin-receptor kinase (*NTRK*); the V600E mutation of the *BRAF* gene (*BRAF^V600E^*); and high tumor mutational burden, high microsatellite instability, and gene mismatch repair-deficient (TMB-H/MSI-H/dMMR) tumors. Ongoing trials investigate HER2, *RET*, and non-*BRAF^V600E^* mutations in CCA and improvements in the efficacy and safety of new targeted treatments. This review aims to present the current status of molecularly matched targeted therapy for advanced CCA.

## 1. Introduction

In the past two decades, molecular targeted and immune therapy has revolutionized the treatment landscape of several malignancies. Recent findings highlighted that matched targeted therapy improved the response rate and prolonged the survival of patients with advanced cancers [1,2]. Some of the most notable achievements are in non-small cell lung cancer (NSCLC), a disease for which the number of actionable biomarkers has increased rapidly. Indeed, NSCLC is now almost a poster child for the benefits of precision medicine, with almost 70% of NSCLCs having a biomarker-based therapy (including but not limited to *EGFR* and *ERBB2* alterations, mismatch repair gene defects, high tumor mutational burden, *BRAF V600E*, *NTRK* fusions, *ALK* fusions, *ROS1* fusions, and *RET* alterations) [3,4,5,6,7,8,9,10]. Moreover, molecular diagnostics have supported the development of drugs and therapeutic antibodies targeting specific receptors, antigens, or molecular pathways crucial for tumor cell proliferation and invasion, tumor growth, immunity, and metastases across various malignancies [11]. 

Cholangiocarcinoma (CCA) is a promising candidate for targeted therapy due to its diverse molecular features [12]. The incidence of CCA is low in the Western world, with between 0.35 and 2 cases per a 100,000 population per year [13]. The global incidence of CCA has steadily increased over the last 30 years, from 0.1 to 0.6 cases per a 100,000 population [13]. CCA is a highly aggressive malignancy, with a 5-year OS for locally advanced or metastatic disease of less than 10% [14]. CCA arises from the intrahepatic and extrahepatic biliary epithelium [15]. Anatomically, 60–70% of cases are classified as perihilar CCA (pCCA); 20–30% of cases are distal CCA (dCCA); and 5–10% of cases are intrahepatic CCA (iCAA) [15]. Current population statistics show an increased prevalence of CCA [15]. In early-stage CCA, the primary treatment includes surgical resection and adjuvant chemotherapy, while systemic chemotherapy is the standard treatment for advanced-stage CCA [16]. Patients with CCA often present with late-stage disease, and the prognosis is poor [16].

**Figure 1:**
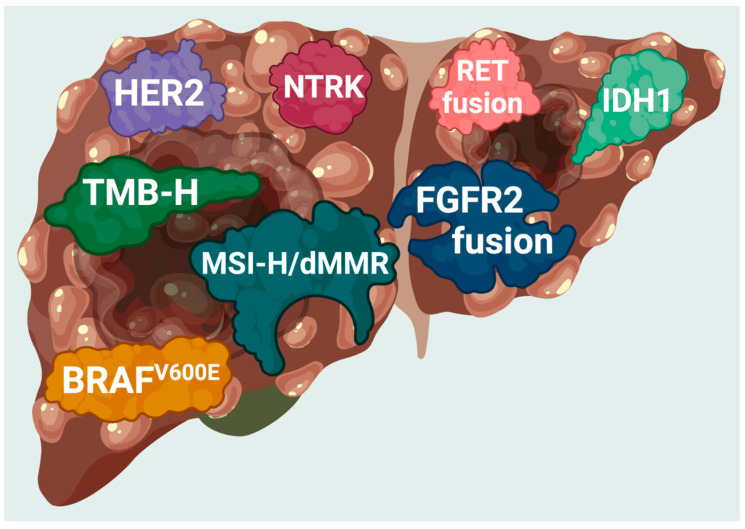
FDA-approved targets found in patients with cholangiocarcinoma. Current genomic targets with FDA-approved therapies in cholangiocarcinoma. MSI-H, microsatellite instability-high; dMMR, deficiency in mismatch repair; TMB-H, tumor mutation burden-high (≥10 mutations/mb).

Molecular techniques, including next-generation sequencing (NGS), have identified novel mutations in tumors from patients with CCA (Figure 1) [17]. Patients with CCA show substantial variations in their molecular profiling and genetic aberrations according to their anatomic locations (Table 1). Promising therapeutic molecular targets for CCA have been identified and include isocitrate dehydrogenases 1 and 2 (*IDH1* and *2*) and products of fusions of the fibroblast growth factor receptor 2 (*FGFR2*) gene [17]. In 2019, the US Food and Drug Administration (FDA) granted accelerated approval for pemigatinib, an *FGFR2* inhibitor, as the first targeted therapy for locally advanced or metastatic iCCA with *FGFR2* fusions or rearrangement [18]. Another approval for a targeted therapy specifically for CCA was on 28 May 2021 [19]. The FDA granted accelerated approval to infigratinib, a kinase inhibitor approved for adults with previously treated, locally advanced, unresectable, or metastatic CCA with an *FGFR2* gene fusion or rearrangement [19]. This specific approval for CCA required confirmation that an FDA-approved diagnostic test detected *FGFR2* gene fusion or rearrangement in CCA [19]. More recently, the FDA granted accelerated approval to futibatinib, an irreversible *FGFR1-4* Inhibitor, for previously treated, locally advanced, unresectable, or metastatic CCA with an *FGFR2* gene fusion or rearrangement on 30 September 2022 [20]. In support of the recent developments in targeted therapies, in August 2021, the FDA approved ivosidenib as a targeted therapy for adult patients with unresectable locally advanced or metastatic CCA with a mutation in the *IDH1* gene detected by an FDA-approved diagnostic test [21]. Finally, *ALK* and *ROS1* mutations occur in between 3% and 9% of patients with CCA, and ALK-positive and ROS1-positive CCA may also be treated with ALK inhibitors [22]. 

There are also several tumor-agnostic approvals that encompass CCA. For example, in May 2017, the FDA granted accelerated approval for the therapeutic monoclonal antibody, pembrolizumab, for microsatellite instability-high (MSI-H) or mismatch repair deficient (dMMR) unresectable or metastatic solid tumors that have progressed despite prior treatment [36]. In 2020, pembrolizumab received FDA approval for adults and children with high tumor mutational burden (TMB-H) solid tumors [37]. In August 2021, accelerated approval was granted for dostarlimab for adult patients with recurrent or advanced solid tumors identified as mismatch repair deficient (dMMR) as determined by an FDA-approved diagnostic test [38]. In 2018 and 2019, two therapies targeting neurotrophic tyrosine receptor kinase (*NTRK*) gene fusion, entrectinib and larotrectinib, were approved for locally advanced or metastatic solid tumors [39,40]. In June 2022, the FDA approved dabrafenib combined with trametinib to treat unresectable or metastatic solid tumors with a *BRAF^V600E^* mutation [41]. 

This review aims to present and discuss the advances in molecular targeted therapy for patients with advanced CCA.

## 2. The Options for Molecular Targeted Therapy

### 2.1. Neurotrophic Tyrosine Receptor Kinase (NTRK) Gene Fusion-Positive Cholangiocarcinoma (CCA)

Molecular profiling of solid tumors has identified clinically actionable fusions of the *NTRK1*, *NTRK2*, and *NTRK3* genes, which encode neurotrophic tropomyosin receptor kinase (NTRK) [42]. *NTRK* fusion products activate the TRK gene and, subsequently, the downstream signaling pathways, PI3K and MAPK, leading to tumor cell proliferation and invasion [43,44]. Therefore, *NTRK* inhibitors are promising targeted therapies for patients with *NTRK* fusion-positive cancers and have shown antitumor responses in NCSLC, melanoma, and other tumors [43,44]. *NTRK* gene fusions have been identified in 1–3% of patients with CCA [45]. Table 2 shows the results of pivotal clinical trials that assessed the outcomes of *NTRK* inhibitors in *NTRK* fusion-positive metastatic or unresectable locally advanced solid tumors.

Larotrectinib is a first-generation, highly selective pan-*NTRK* competitive inhibitor that suppresses cancer cell proliferation [62]. It has shown immediate, robust, and long-lasting anticancer efficacy in pediatric and adult patients with solid tumors harboring TRK fusions [49]. Drilon and colleagues conducted a phase 1 trial in adults (NCT02122913), a phase 1/2 trial in children (NCT02637687), and a phase 2 trial in children and adults (NCT02576431) with locally advanced or metastatic solid tumors who had received previous standard systemic therapy and were then given larotrectinib (100 mg twice daily), see Table 1 [49]. A total of 55 patients with 17 TRK fusion-positive tumor types included two patients (4%) with CCA [49]. The objective response rate (ORR) was high, at 80% (95% CI, 67–90) [49]. At one year, 55% of patients were still progression-free [49]. The median duration of response (MDR) and the progression-free survival (PFS) remain unmet [49]. The most common toxicities ≥ grade 3 included a raised ALT or AST level (9%), anemia (3.6%), reduced neutrophil count (3.6%), and nausea (3.6%) [49]. Based on these findings, larotrectinib was granted accelerated approval by the FDA in November 2018 for adult and pediatric patients with *NTRK*-positive solid malignant tumors, either metastatic or where surgical resection is unfeasible due to severe morbidity, who have progressed on systematic therapy [39]. Larotrectinib is also approved for patients with no satisfactory alternative treatments [39]. Currently, the MD Anderson Cancer Center and the National Cancer Institute (NCI) are conducting a phase 2 trial to investigate the efficacy of larotrectinib in previously treated patients with locally advanced or metastatic solid tumors and *NTRK* gene amplification (NCT04879121). Another ongoing phase 2 trial aims to assess the efficacy of larotrectinib in pediatric patients with relapsed or refractory advanced solid tumors with *NTRK* gene fusion (NCT03213704), Table 3.

Entrectinib is another selective pan-TRK inhibitor with activity against ROS1 and ALK [63,64]. In two phase I studies (ALKA-372–001 and STARTRK-1), entrectinib was administered to 119 patients with relapsed or refractory advanced/metastatic solid tumors harboring *NTRK*1/2/3, ROS1, or ALK gene fusions [47]. Entrectinib was well-tolerated and only 15% of patients required a dose modification [47]. The most common grade ≥ 3 toxicities included fatigue/asthenia (4%), weight increase (2%), diarrhea (1%), and eosinophilic myocarditis (1%), Table 2 [47].

The phase 2 STARTRK-2 trial was an open-label, multicenter, global basket study that included patients with solid tumors harboring *NTRK1/2/3, ROS1*, or *ALK* gene fusions (NCT02568267). A focused integrated analysis on *NTRK* fusion-positive tumors showed that at a median follow-up of 12.9 months (interquartile range (IQR), 8.77–18.76), the median duration of response was 10 months (95% confidence interval (CI), 7.1–not reached) and the objective response rate (ORR) was 57% (95% CI, 43.2–70.8) [48]. The median overall survival (OS) was 21 months (95% CI, 14.9–NE) and the median progression-free survival (PFS) was 11.2 months (95% CI, 8.0–14.9) [48]. Major adverse events (≥3 grade) were reported in 61.6% of patients and included anemia (12%), an increase in weight (10%), and fatigue (7%), with no patient mortality, Table 2 [48]. Based on these findings, entrectinib gained accelerated approval by the FDA in August 2019 at a dose of 600 mg once daily [40]. Approval was for use in patients with *NTRK* gene fusion and metastatic or unresectable locally advanced solid tumors, who have progressed on systemic therapy or have no satisfactory alternative treatment [40]. 

The approval of entrectinib provides an additional treatment option for patients with advanced cancer, potentially creating the opportunity for patients and their physicians to choose between different therapies [65]. Larotrectinib is available in a liquid formulation approved for children younger than 12 years old, whereas entrectinib is not. However, entrectinib may be effective for children with brain tumors, while the efficacy of larotrectinib for primary and metastatic brain tumors is currently being evaluated [65]. The two drugs also have different side effect profiles. The adverse events seen most frequently in the larotrectinib trials include increased ALT or AST, fatigue, and vomiting. Warnings and precautions for larotrectinib include neurotoxicity, hepatotoxicity, and embryo–fetal toxicity. Entrectinib has additional warnings and precautions, including congestive heart failure, CNS effects, and skeletal fractures [65]. The National Comprehensive Cancer Network (NCCN) guidelines recommend larotrectinib and entrectinib as first-line or subsequent-line (following disease progression) treatment options for unresectable or metastatic iCCA and eCCA with *NTRK* gene fusions. Both entrectinib and larotrectinib are approved in the United States and Europe for the treatment of unresectable or metastatic solid tumors with *NTRK* gene fusion and progression after previous therapy [66,67]. However, there are currently limited data for patients with CCA.

### 2.2. Cholangiocarcinoma (CCA) with BRAF^V600E^ Mutations

Mitogen-activated protein kinase (MAPK) signaling is essential for cell growth and survival through the RAS/RAF/MEK/ERK pathway [68]. The *BRAF* gene is an oncogene whose protein product upregulates the RAS/RAR/MEK pathway [68]. *BRAF* mutations have been identified in several solid malignancies, including colorectal cancer, NSCLC, and melanoma [69,70]. More than 50 different *BRAF* mutations have been reported, with the V600E point mutation being the most common mutation (*BRAF^V600E^*) [71]. In *BRAF^V600E^*, valine (V) is substituted by glutamic acid (E) at amino acid 600, resulting in activating *BRAF* with subsequent tumor growth and spread [72]. In CCA, *BRAF* mutations are uncommon, occurring almost exclusively in iCCAs, with a prevalence ranging between 5% and 7%, and with the *BRAF^V600^*^E^ mutation in 1.5% of patients with iCCA [26,73].

Dabrafenib is a competitive inhibitor of the RAF protein, which causes apoptosis by decreasing downstream phosphorylation of MEK and ERK, arresting the cell cycle in G1, and activating caspase-3/7 [74,75]. Even if a tumor initially responds to dabrafenib alone, it may eventually become resistant to treatment if another pathway activates the MEK protein [75]. Trametinib is a selective inhibitor of MEK1/MEK2 and is used with dabrafenib, which prevents tumors from using this escape mechanism [76]. Trametinib reduces cell proliferation, causes G1 cell-cycle arrest, and induces apoptosis [77]. The combination of these drugs in targeting MEK and *BRAF* has yielded promising results (Table 2). In the phase 2, single-arm, open-label trial ROAR, the *BRAF* cohort included 43 adult patients with *BRAF^V600E^*-mutated CCA with metastatic, locally advanced, unresectable, or recurrent disease that had progressed on prior therapy [50]. Patients received trametinib 2 mg once daily and dabrafenib 150 mg twice daily, with a mean follow-up of 10 months [50]. The ORR was 51% (95% CI, 36–67), the mean OS was 14 months (95% CI, 10–33), the mean PFS was 9 months (95% CI, 5–10), and the MDR was 9 months (95% CI, 6–14) [50]. Increased gamma-glutamyl transferase (GGT) (12%), low WBC count (7%), and pyrexia (7%) were the most common grade ≥ 3 adverse events, Table 2 [50]. 

Salama et al. reported results from the NCI-MATCH trial, a single-arm, open-label study that enrolled 29 patients with different solid tumors that progressed on standard lines of therapy, including four patients with iCCA [51]. Patients were given a continued dosing of dabrafenib (150 mg twice a day) and trametinib (2 mg once daily) [51]. The ORR was 38% (90% CI, 22.9 –54.9%), the median OS was 28.6 months, the median PFS was 11.4 months (90% CI, 8.4–16.3), and the median MDR was 25.1 months (90% CI, 12.8–NE) [51]. Three of the four patients with CCA had a partial response and grade ≥ 3 adverse events occurred in 65.7% of the enrolled patients [51]. Fatigue (11.4%), decreased neutrophil count (8.6%), and decreased WBC count (8.6%) were the most frequently reported grade ≥ 3 adverse events associated with the treatment, Table 2 [51]. The results of these trials supported the FDA approval of the combination of dabrafenib and trametinib for treating adults and children >6 years with *BRAF^V600E^* mutation-positive, unresectable, or metastatic solid tumors who have progressed on prior therapy [41]. 

#### Other *BRAF* Inhibitors Currently Undergoing Clinical Trials

In a phase 1 study, patients with *BRAF^V600^*-mutated solid tumors (including CCA) are currently being evaluated for a response to ABM-1310, a selective inhibitor of *BRAF^V600E^* mutation tumors (NCT04190628). Patients with advanced solid tumors, including biliary tract cancers, with *BRAF* mutations, are the focus of a phase 1 study of BGB-3245, a second-generation *BRAF* inhibitor (NCT04249843), Table 3. Combining the selective ERK1/2 inhibitor JSI-1187 with a *BRAF* inhibitor is another potential study strategy.

Despite the significant advances in managing patients with *BRAF^V600^* mutations, further studies are required. For example, in studying the efficacy of dabrafenib and trametinib and concurrent mutations of *TP53* and *BRAF^V600E^*, early studies reported that this was associated with a more aggressive disease, resulting in less clinical benefits from dabrafenib and trametinib [78]. In addition, patients with *BRAF^V600E^/TP53* mutations were associated with reduced PFS and OS [79].

### 2.3. Cholangiocarcinoma (CCA) with Fibroblast Growth Factor Receptor 2 (FGFR2) Gene Fusion or Rearrangement

Alterations in the FGFR gene and dysregulated FGFR signaling play a role in the development and progression of several types of cancer, including CCA. Four receptors belong to the FGFR family, including *FGFR1*, *2, 3*, and *4*, which share a cytoplasmic tyrosine kinase domain [80]. *FGFR2* alterations include rearrangements, amplifications, and mutations, present in 10–16% of patients with iCCA [33]. These alterations activate mitogen-activated protein kinases (MAPKs), triggering constitutive signaling cascades that prompt tumor cell proliferation, survival, migration, and angiogenesis [81,82]. Therefore, FGFR inhibitors are promising targeted therapies that can potentially improve the survival of patients with CCA. Initially, non-selective tyrosine kinase inhibitors (TKIs) were investigated in phase 1/2 clinical trials and showed low antitumor activity and a limited survival benefit [83,84,85]. More recently, selective FGFR inhibitors were introduced for patients with *FGFR2* fusion-positive iCCA and resulted in a significant clinical response, prompting phase 2/3 trials and accelerated FDA approvals [18].

Pemigatinib is a selective inhibitor of *FGFR1*, *2*, and *3* that competitively inhibits the autophosphorylation and FGFR-mediated signaling cascades in tumor cells [86]. In the phase 2, multicenter, open-label FIGHT-202 trial, previously treated patients with metastatic CCA with *FGFR2* fusions or *FGFR2* rearrangements (*n* = 107), other *FGFR* mutations (*n* = 20), or wild-type FGFR (*n* = 18) received 13.5 mg of pemigatinib once daily on day 1–14 of a 21-day cycle [52]. In patients with *FGFR2* fusions or *FGFR2* rearrangements, the objective response rate (ORR) was 35.5% (95% CI, 26.5–45.4%) [52]. The median PFS and OS were 6.9 and 21.1 months, respectively [52]. Up to 64% of the patients had grade ≥ 3 toxicities that included hyper/hypophosphatemia (12%), arthralgia (6%), fatigue (5%), and retinal detachment (4%), Table 2 [52]. Based on these results, pemigatinib became the first FDA-approved targeted therapy for previously treated metastatic CCA with *FGFR2* fusions or *FGFR2* rearrangements [18]. Currently, FIGHT-302 is an ongoing phase 3 clinical study comparing pemigatinib with gemcitabine and cisplatin chemotherapy to determine the drug’s efficacy in the first-line treatment of CCA (NCT03656536), Table 3.

Infigratinib is another highly selective ATP-competitive *FGFR1–3* inhibitor that showed promising antitumor activity in patients with *FGFR2* fusions in early-phase trials [87]. Patients with advanced iCCA resistant to chemotherapy were enrolled in a phase 2 study of infigratinib in a multicenter, open-label, phase 2 trial [53]. Of the 122 enrolled patients, 108 had positive *FGFR2* fusions or *FGFR2* rearrangements [53]. Patients received 25 mg once daily infigratinib for 21 days in a 28-day cycle [53]. In patients with *FGFR2* fusions or *FGFR2* rearrangements, the ORR was 23.1% (95% CI, 15.6–32.3%), indicating clinically significant activity after treatment [53]. In this trial, one patient had a complete response (CR) and 24 patients had partial responses [53]. The median duration of response (DOR) was five months (95% CI, 3.7–9.3), and eight patients had a maintained response for more than six months [53]. Two-thirds of the patients (66.2%) had grade ≥ 3 toxicities, with hypophosphatemia (14.1%) and hyperphosphatemia (12.7%) being the most common adverse events, Table 2 [53]. A phase 1 dose-escalation study showed that the risk of hyperphosphatemia in patients receiving infigratinib increased with higher drug exposure and was associated with higher antitumor activity [88]. On May 2021, the FDA granted accelerated approval to infigratinib for previously treated locally advanced CCA or for patients with metastatic CCA with *FGFR2* fusions or *FGFR2* rearrangements [19].

Infigratinib is also a potential targeted therapy for patients with untreated CCA with *FGFR2* fusions or *FGFR2* rearrangements. PROOF-301 is an ongoing phase 3 trial that recruited patients with untreated locally advanced CCA or patients with metastatic CCA with *FGFR2* fusions or *FGFR2* rearrangements [89]. In PROOF-301, patients received either infigratinib or a standard chemotherapy regimen of gemcitabine and cisplatin [89]. Infigratinib may potentiate the apoptotic activities of chemotherapies in multidrug-resistant tumor cells [90]. Therefore, there is a potential future role for infigratinib in combination therapy for patients with advanced CCA, and the results of further clinical trials are awaited with interest.

Futibatinib is an irreversible *FGFR1-4* inhibitor that binds to the conserved cysteine residues of the P-loop of the kinase domain [91]. Previous studies showed that futibatinib exhibited highly selective antitumor activities against tumor cells harboring FGFR mutations, particularly against mutations commonly associated with resistance to ATP-dependent FGFR inhibitors [91]. In addition, the oral futibatinib molecule was associated with a comparatively lower number of drug-resistant clones than ATP-dependent FGFR inhibitors [91,92]. Futibatinib was studied in a phase I trial that recruited 197 patients with previously-treated advanced solid tumors, 83 of which had CCA with a mutation, fusion, or amplification of *FGFR2* (NCT02052778) [92]. In this trial, 64 patients were treated with futibatinib at 20 mg and 19 patients at 16 mg [92]. The results showed that futibatinib at 20 mg daily resulted in an ORR of 15.6%, a disease control rate (CDR) of 71.9%, a median DOR of 5.3 months, and a median PFS of 5.1 months [92]. The updated analysis of the single-arm FOENIX-CCA2 phase 2 trial included 103 patients with previously-treated advanced or metastatic iCCA with *FGFR2* fusions or *FGFR2* rearrangements (NCT02052778) [54]. Futibatinib 20 mg daily led to an ORR of 41.7% at a median follow-up period of 25.0 months [54]. The DC was 82.5%, the median PFS was 8.9 months, and the median OS was 20.0 months, Table 2 [54]. Based on these findings, on September 30, 2022, the FDA granted accelerated approval for futibatinib for adult patients with previously treated locally advanced or metastatic iCCA with *FGFR2* gene fusion or rearrangement [20].

Derazantinib is another potent anti-*FGFR1-3* that showed promising antitumor activity in iCCA harboring *FGFR2* fusions or *FGFR2* rearrangement. In the phase 2 FIDES, 143 patients with iCCA harboring *FGFR2* fusions (*n* = 103) or *FGFR2* mutations or amplifications (*n* = 40) received derazantinib 300 mg once daily [93]. In the cohort with *FGFR2* fusions, the ORR was 21.4% (95% CI 13.9–30.5), with a median PFS and OS of 8.0 (95% CI 5.5–8.3) and 17.2 (95% CI 12.5–22.4) months, respectively [93]. In the *FGFR2* mutations or amplifications cohort, the ORR and DCR were 6.5% (95% CI 0.8–21.4) and 58.1% (95% CI 39.1–75.5). The median PFS was 8.3 (95% CI 1.9–16.7) and the median OS was 15.9 (95% CI 8.4–not estimated) [93]. The most common grade ≥ 3 adverse events in the overall cohort were hyperphosphatemia (3%), asthenia/fatigue (5%), nausea (1%), and transaminase elevations (12%) [93].

The phase 1/2 ReFocus trial evaluated RLY-4008, a selective *FGFR2* inhibitor that can target FGFR resistance mutations, in CCA patients with *FGFR2* fusions or *FGFR2* rearrangements who did not receive FGFR inhibitors before. The preliminary analysis of 38 patients showed an ORR of 63.2% (95% CI 46.0–78.2) and a DCR of 94.7%. There was no observation of grade 4/5 adverse events [94].

Despite the promising findings of selective FGFR inhibitors in patients with CCA and *FGFR2* fusions or *FGFR2* rearrangements, several issues remained unanswered. More than 150 fusion partners are associated with *FGFR2* gene rearrangement, which results in significant molecular diversity in patients with *FGFR2* fusions and rearrangements [95,96]. Furthermore, nearly 50% of the gene fusion and rearrangement partners are present within the same chromosome of the *FGFR2* gene [95,96]. 

It is still unclear which patients might respond to *FGFR2*-targeted therapies and whether fusion partners can affect the response and survival with *FGFR2*-targeted therapies [96]. Therefore, future research should focus on the effect of combined genetic alterations on the responses to *FGFR2* inhibitors and their role in the development of acquired resistance, as well as identifying reliable response biomarkers for *FGFR2*-targeted therapies [96,97].

### 2.4. High Tumor Mutational Burden (TMB-H) as a Predictive and Prognostic Biomarker

TMB is a recently identified biomarker of the response to immune checkpoint inhibitors (ICIs) in several types of cancer [97]. The TMB is the number of somatic mutations per megabase (Mb) of the genomic sequence of a tumor [97]. A TMB score of ≥10 mutations/Mb has been proposed as a threshold with a high likelihood of neoantigen formation and represents TMB-H status [97]. In patients with several tumor types, including melanoma, NSCLC, and bladder cancer, patients with TMB-H had better outcomes when treated with programmed death protein-1 and programmed cell death ligand-1 (PD-1/PD-L1) checkpoint inhibitors, or a cytotoxic T lymphocyte antigen 4 (CTLA-4) blockade [98,99,100]. TMB-H has been detected in 27.3% of patients with iCCA [101]. 

Pembrolizumab is a humanized antibody that inhibits the PD-1 receptors on lymphocytes by inhibiting the ligands that would block the receptor and inhibit an immune response [102,103,104]. In a subgroup analysis of 102 patients with TMB-H, who were enrolled in the phase 2 KEYNOTE-158 trial and received pembrolizumab, the ORR was 29% (95% CI, 21–39%), the median OS was 11.7 months (95% CI, 9.1–19.1), and the median PFS was 2.1 months (95% CI, 2.1–4.1) [105]. Notably, there were no patients with CCA in the TMB-H subgroup of this trial [105]. However, based on these findings, in 2017, the FDA approved pembrolizumab to treat adult and pediatric patients with unresectable or metastatic solid tumors (including CCA) [36]. The indications for this approval also required that the tumor tissue was TMB-H (≥10 mutations/megabase), and that the patients had progressed following prior therapy and for whom there were no satisfactory alternative treatment options [36]. 

However, it is unclear if the TMB levels for predicting the response to the PD-1 blockade are consistent throughout the spectrum of solid tumors. There are situations in which a high TMB does not indicate a response. Therefore, novel biomarkers are required that reflect the complexity of the tumor immune microenvironment and consider the effects of tumor mutations on the immune response.

### 2.5. High Microsatellite Instability and Mismatch Repair Deficient (MSI-H/dMMR) Cholangiocarcinoma (CCA)

The production of neoantigens and CD8+ T cell infiltrations into the tumor microenvironment are both increased in tumors with dMMR or high levels of MSI [106]. MSI-H/dMMR makes the errors produced during DNA replication difficult to repair, which leads to mutations [106]. The prevalence of MSI-H/dMMR in patients with iCCA ranges from 4.7–18.2% [35]. 

As part of the phase 2 KEYNOTE-158 study, pembrolizumab 200 mg intravenously once every three weeks was administered to 233 patients with advanced solid tumors and confirmed MSI-H/dMMR [56]. In this study, 22 (9.4%) patients had CCA [56]. With a median follow-up of 13.4 months, the ORR was 34.3% (95% CI, 28.3–40.8%), the median OS was 23.5 months (95% CI, 13.5–NR), and the median PFS was 4.1 months (95% CI, 2.4–4.9 months [56]. The median duration of the response was not reached (range, 2.9–31.3 months) [56]. A subgroup analysis of CCA showed that the ORR was 40.9% (95% CI, 20.7–63.6), the median OS was 24.3 months (95% CI: 6.5–NE), and the median PFS was 4.2 months (95% CI, 2.1–NE) [56]. Adverse events ≥ grade 3 occurred in 34 patients (14.6%) [56]. Pneumonitis (1.3%), severe skin reactions (1.3%), and colitis (0.9%) were the most common adverse events, Table 2 [56].

In an open-label, single-arm, phase 2 clinical trial, 86 patients with 12 types of cancer, including four patients with CCA, with at least one prior cancer therapy and MSI-H/dMMR mutations, were included [55]. The patients received 10 mg/kg of pembrolizumab every 14 days [55]. The ORR was 53% (95% CI, 42–64%), the 2-year OS was 64% (95% CI, 53–78%), and the 2-year PFS was 53% (95% CI, 42–68%) [55]. A subgroup analysis of patients with CCA showed that the ORR was 50%, the CR was 25%, the SD was 75%, and the disease control rate was 100% [55]. Minor adverse events were reported in 20%, with the most common being diarrhea/colitis (6%), pancreatitis/hyperamylasemia (6%), fatigue (2%), and anemia (2%), Table 2 [55]. Based on these findings, in 2020, the FDA approved the use of pembrolizumab in adults and pediatric patients who have unresectable or metastatic MSI-H/dMMR solid tumors that have progressed despite prior treatment, without satisfactory alternative treatment options [37]. The results of other trials for MSI-H patients in solid tumors are awaited. For example, a phase 1/2 trial of pevonedistat, a selective NEDD8 inhibitor, in combination with pembrolizumab in patients with dMMR/MSI-H solid cancers is ongoing (NCT04800627), Table 3.

Dostarlimab is another anti-PD-1 monoclonal antibody [107]. The phase 1 GARNET study was a non-randomized, multicenter, open-label trial to evaluate the safety and efficacy of dostarlimab (500 mg every 3 weeks for four cycles, then 1000 mg every 6 weeks) in 106 patients with advanced solid tumors (two of whom had CCA) with confirmed MSI-H/dMMR, one of whom had a biliary tract cancer [57]. The ORR was 38.7% (95% CI, 29.4–48.6) [57]. With a median duration of follow-up of 12.4 months, the median DOR was not reached [57]. At 12 months, the Kaplan–Meier estimate for the chance of response preservation was 91%, and at 18 months it was 80% [57]. Approximately 8.3% of patients reported at least one grade 3 adverse event, including anemia (3.9%), elevated lipase (2.3%), elevated ALT (1.6%), and diarrhea (1.6%), Table 2 [57]. Based on the findings from this study, the FDA approved dostarlimab in adult patients with MSI-H/dMMR recurrent or advanced solid tumors that progressed on or following prior treatment and were not candidates for satisfactory alternative treatment options [38].

Given the limited single-agent activity and the limited number of patients with CCA included in these clinical trials, further studies are required to investigate novel immunotherapy combinations to enhance the treatment efficacy in patients with CCA. 

### 2.6. Isocitrate Dehydrogenase Isoenzyme (IDH1) Gene Mutations in Cholangiocarcinoma (CCA)

*IDH1* gene mutations commonly occur in CCA [108]. Missense mutations in the *IDH1* R132 codon leads to the overproduction of the oncometabolite R-2-hydroxyglutarate (R-2HG) [109]. In tumor progenitor cells, an increase in the R-2HG levels inhibits cellular differentiation and drives oncogenesis by promoting histone methylation and DNA methylation [110]. The prevalence of an *IDH1* mutation in iCCA has been estimated at 13% [34]. 

Ivosidenib is a small molecule inhibitor of *IDH1*. In a phase 1, multicenter, open-label study, 73 patients with *IDH1*-mutant CCA (89% iCCA and 11% eCCA), refractory to other systemic therapy, were enrolled and received ivosidenib (200–1200 mg daily in 28-day cycles) [58]. The ORR was 5% (95% CI, 1.5–13.4), the median OS was 13.8 months (95% CI, 11.1–29.3), and the median PFS was 3.8 months (95% CI, 3.6–7.3) [58]. Approximately 23% of the included patients had grade ≥ 3 adverse events, including ascites (5%), anemia (4%), and fatigue (3%), Table 2 [58]. This trial led to a multicenter, randomized, double-blind, placebo-controlled phase 3 study (ClarIDHy), which included 185 adult patients with advanced CCA with *IDH1* mutations who had progressed on previous therapy [34]. Patients were randomly assigned to oral ivosidenib 500 mg or matched placebo once daily in continuous 28-day cycles [34]. In the intervention group, with a median follow-up of 6.9 months, the ORR was 2% (95% CI, 0.5–6.9), and the median PFS was significantly improved (median PFS 2.7 months; 95% CI, 1.6–4.2) [34]. The median OS after accounting for the cross-over was 10.3 months (95% CI, 7.8–12.4), which was significantly better than the placebo (median OS = 5.1 months) [34]. Grade ≥ 3 adverse events were reported in 30% of the patients [34]. The most frequently reported adverse event grades ≥ 3 were ascites (7%), increased AST (5%), anemia (3%), and fatigue (3%), Table 2 [34]. Based on this trial, the FDA approved ivosidenib for the treatment of adult patients with unresectable locally advanced or metastatic *IDH1*-mutated CCA who had been previously treated [21]. 

PARP inhibitors are also being studied in this subgroup of patients as they exhibit dysregulated homologous recombination repair. The National Cancer Institute (NCI) is conducting a phase 2 clinical trial that aims to investigate the safety and efficacy of olaparib as a subsequent line therapy for patients with advanced solid tumors (including CCA) with *IDH1* or *IDH2* mutations (NCT03212274). Another ongoing study combined ceralasertib and olaparib in patients with refractory CCA and advanced solid tumors with *IDH1/2* (NCT03878095), Table 3.

The use of *IDH1* inhibitors as single treatments has shown promising results in targeted therapy of malignancies harboring *IDH1* mutations in preclinical and clinical settings. However, these studies are preliminary and currently include small numbers of patients with CCA.

### 2.7. Erb-B2 Receptor Tyrosine Kinase 2 (ERBB2)/Human Epidermal Growth Factor Receptor 2 (HER2)-Positive Cholangiocarcinoma (CCA)

The *ERBB2* gene encodes HER2, a receptor tyrosine kinase found in the plasma membrane [111]. *ERBB2* triggers several signaling pathways involved in tumor growth [112]. Tumorigenesis is associated with HER2 and MAPK pathway dysregulation [32,113]. The overexpression of HER2 has been reported in 5.8% of iCCA and 13–20% of eCCA [31,32]. 

Pertuzumab and trastuzumab are monoclonal antibodies that target HER2 and are used to treat HER2-positive malignancies [114]. The combination of pertuzumab and trastuzumab suppresses HER2-AKT signaling by inhibiting both the ligand-induced and ligand-independent HER2-HER3 complex formations [114]. A phase 2, single-arm, multicenter trial called MyPathway included 39 patients with previously treated HER2-positive metastatic CCA [59]. Patients were treated with pertuzumab (840 mg loading dose and 420 mg every three weeks) plus trastuzumab (8 mg/kg loading dose, 6 mg/kg every three weeks) [59]. The ORR was 23% (95% CI, 11–39), the median PFS was 4.0 months (95% CI, 1.8–5.7), and the MDR was 10.8 months (95% CI, 0.7–25.4) [59]. Grade ≥3 adverse events were reported in 46% of patients, with the most common being an increase in the ALT and AST (13%) and an increase in ALP (10%), Table 2 [59]. These were promising results for patients with HER2-mutated CCA. However, regulatory approval for patients with CCA is awaited.

More recently, trastuzumab deruxtecan (T-DXd) showed promising antitumor activity in HER2-positive advanced solid tumors, including CCA. In a phase 1 trial of 60 patients with advanced, non-breast/non-gastric, HER2-mutant solid tumors (NCT02564900), the ORR was 28.3% and the median PFS was 7.2 (95% CI 4.8–11.1) months [115]. The phase 2 HERB trial recruited 32 patients (24 with HER2-positive and eight with HER2-low) BTCs who received T-DXd. The efficacy cohort included 22 patients (9 had CCA). In patients with HER2-positive BTCs, the ORR was 36.4%, the median PFS was 4.4 months, and the median OS was 7.1 months. In the HER2-low group, the ORR, median PFS, and median OS were 12.5%, 4.2 months, and 8.9 months, respectively. The rate of grade ≥ 3 adverse events was 81.3% [116].

Several ongoing studies aim to investigate targeted HER2 agents in treating patients with CCA with *ERBB2* mutations (Table 3). In the front-line setting, gemcitabine combined with cisplatin and trastuzumab and the combination of gemcitabine, cisplatin, and varlitinib are currently being studied in two clinical trials (NCT03613168 and NCT02992340). In addition, HER2-targeting agents are currently being studied for subsequent lines of therapy, either as monotherapy or in combination with standard chemotherapy agents. Ongoing trials include: the oral HER2 covalent inhibitor, TAS0728, (NCT03410927); the HER2 antibody-drug conjugate, trastuzumab deruxtecan (NCT04482309 and JMA-IIA00423); the antibody-drug conjugate, RC48-ADC, (NCT04329429); capecitabine plus the dual HER2 and *EGFR* inhibitor, varlitinib (NCT03093870); chemotherapy (5-FU or IRI or Cape) plus trastuzumab (NCT03185988); and chemotherapy plus the HER2-targeted bispecific antibody, zanidatamab (NCT02892123). 

The results of these ongoing trials may provide multiple options for targeted treatments for patients with CCA in the molecular subgroup and improve patient survival. 

### 2.8. RET Gene Fusion-Positive Cholangiocarcinoma

More than three decades have passed since the discovery of the gene encoding the receptor tyrosine kinase, *RET* [117]. *RET* rearrangements and mutations are recognized as treatable drivers of oncogenesis [117]. Certain *RET* fusion proteins and activating point mutations can drive oncogenesis and tumor progression by activating downstream signaling pathways, leading to uncontrolled cell proliferation [117]. *RET* gene fusions are present in between 1% and 2% of NSCLC and thyroid cancers and represent potential targets for therapeutic inhibition of *RET* kinase [118]. However, *RET* fusions seem rare in CCA, and data regarding their exact prevalence in CCA are limited [119]. 

Pralsetinib is a selective inhibitor of the *RET* receptor tyrosine kinase. In the phase 1/2, open-label ARROW trial, 29 patients with *RET* fusion-positive solid tumors were included, with three patients with CCA [60]. Patients received a starting dose of pralsetinib of 400 mg QD [60]. The ORR was 57% (95% CI, 35–77%), the median OS was 13.6 months (95% CI, 7.5–NE), the median PFS was 7.4 months (95% CI, 5.1–13.6), and the median duration of response was 11.7 months (95% CI, 5.5–19.0) [60]. Altogether, 69% of patients had grade ≥ 3 adverse events that included neutropenia (31%), anemia (14%), and increased AST (10%), Table 2 [60]. Based on the findings of this trial, the FDA approved pralsetinib for adult and pediatric patients with advanced or metastatic *RET*-fusion-positive lung and thyroid cancers for whom systemic therapy is indicated [120].

Selpercatinib is another highly selective inhibitor of the *RET* receptor tyrosine kinase, with CNS activity [121]. In the phase 1/2, open-label LIBRETTO-001 trial, 45 patients with *RET* fusion-positive solid tumors other than lung or thyroid tumors were included, with two patients with CCA [61]. Of the 45 patients, 43 received a starting recommended dose of 160 mg BID. In the 41 patients who were evaluated for efficacy, the ORR was 43.9% (95% CI, 28.5–60.3), the median OS was 18.0 months (95% CI, 10.7– NE), the median PFS was 13.2 months (95% CI, 7.4–26.2), and the median duration of response was 24.5 months (95% CI: 9.2–NE). One patient with CCA was evaluated for efficacy; the ORR was 100% and the duration of response was 5.6 months [61]. Altogether, 49% of patients had grade ≥ 3 adverse events that included hypertension (22%), increased ALT (16%), and increased AST (13%), Table 2 [61]. Selpercatinib is currently approved for locally advanced or metastatic *RET*-fusion-positive solid tumors [122].

Again, further studies are needed to focus on CCA to validate these findings in this group of patients.

## 3. Liquid Biopsy for Assessment of Circulating Tumor DNA (ctDNA) and Cholangiocarcinoma (CCA)

Liquid biopsies refer to blood sampling to detect circulating tumor DNA (ctDNA), circulating cell-free RNA (ccfRNA), and cell-free DNA (cfDNA) [123,124]. Liquid biopsy as an adjunctive diagnostic method has gained popularity over the last decade due to its potential benefits for cancer patients [123]. The most commonly interrogated element in liquid biopsies is ctDNA or DNA fragments produced from tumors [125]. Treatment resistance tracking, response monitoring, target selection, relapse detection, and early diagnosis are the potential benefits of identifying tumor-derived material in liquid biopsies [126].

Although liquid biopsy is a potentially useful diagnostic tool for patients with CCA, this modality remains underexplored in CCA. Advances in this diagnostic area for patients with CCA have been limited by several practical factors, including the small amounts of ctDNA shed into the bloodstream in patients with localized tumors [127]. On the other hand, CCA is an internal malignancy and it is often difficult to obtain a tissue biopsy. It is also challenging to acquire sufficient tumor cells for diagnosis on aspiration cytology, and these challenges can prevent adequate molecular tumor profiling for CCA [128]. Therefore, blood-derived ctDNA may play an important role in identifying molecular alterations in patients with CCA who may not have an adequate biopsy or cytology sample available for analysis [129]. Consistent mutation findings between tumor tissue and ctDNA were reported in 2015 by Zill et al. in a prospective study of 26 pancreaticobiliary cancers, including eight patients with CCA [130]. In this preliminary study, 93% of mutations found in tissue samples were also identified by cfDNA [130]. In 2019, Mody et al. analyzed ctDNA from 138 patients with biliary tract cancers and identified genetic alterations in 89% of the samples [131].

Recently, Kumari et al. evaluated the diagnostic role of cfDNA in gallbladder carcinoma [132]. Serum was obtained from 34 patients with gallbladder carcinoma and 39 matched controls without malignancy [132]. This study showed that the cfDNA levels were lower in healthy individuals than in patients with gallbladder cancer [132]. In addition, there was a significant correlation between the cfDNA and the presence of jaundice, lymph node metastases, and overall disease TNM stage [132]. Therefore, the quantitative analysis of cfDNA may have the potential to be a unique marker for the molecular detection of targeted therapies in CCA. Furthermore, the analysis of cfDNA may have a role in distinguishing between neoplastic and inflammatory conditions of the gallbladder and biliary tract identified by imaging but without available biopsy material. However, concerns remain about the overall sensitivity of ctDNA mutations for diagnosing early-stage CCA [133].

An additional feature of ctDNA/cfDNA is to track the development of resistance to chemotherapy and targeted treatments [134]. In 2019, Ettrich and colleagues sequenced 15 common gene mutations in ctDNA samples from patients with biliary tract cancer throughout their chemotherapy [135]. In the iCCA cohort (*n* = 13), there was a 92% agreement between tissue samples and blood-derived ctDNA [135]. The level of agreement for the overall cohort was 74% [135]. A change in the mutational profile was also seen in 63% of chemotherapy-naive individuals after treatment [135]. A study reported in 2017 showed that the integrative genomic analysis of cfDNA could identify acquired treatment resistance due to multiple recurrent point mutations in the *FGFR2* kinase domain during tumor progression [136].

## 4. Conclusions

Until recently, clinical studies considered CCA a homogeneous entity, which may have led to the limited antitumor activity of conventional treatment regimens. It is now apparent that the behavior and responses of CCA vary substantially according to the underlying molecular profile. These relatively recent findings highlight the crucial role of precision medicine in guiding the treatment selection for patients with CCA. 

The number of investigational and approved targeted therapies for CCA has increased exponentially in the past decade. Several targeted agents are now approved as first-line and subsequent treatments for patients with locally advanced or metastatic CCA. The selective FGFR inhibitors pemigatinib and infigratinib and the *IDH1* inhibitor ivosidenib are now approved for previously treated patients with *FGFR2* fusions or *FGFR2* rearrangements and *IDH1* mutations, respectively. These gene fusions, rearrangements, and mutations are present in a subset of patients with iCCA. Pemigatinib and infigratinib are also currently being investigated in the first-line setting. Other targeted therapies are available across solid tumors, including CCA, and include: *NTRK* inhibitors (entrectinib and larotrectinib); a *BRAF*/MEK inhibitor combination (dabrafenib and trametinib); pembrolizumab (for high ≥ 10 mutations/mb tumor mutational burden or mismatch repair defect cancers); and *RET* inhibitors (selpercatinib). Targeted therapies for other genomic alterations in CCA and solid tumors in general continue to be developed and explored.

Further therapeutic possibilities for patients with CCA may emerge as our knowledge of the tumor microenvironment and its impact on tumor growth increases. Combined treatments that target both actionable mutations and the tumor microenvironment is another approach that assists with patient selection for the most appropriate molecular targeted therapy.

The value of blood-derived ctDNA in the clinic is currently being explored in CCA, especially since tissue biopsies can be difficult in this type of cancer. 

## 5. Future Directions

Despite multiple FDA approved targeted therapies for patients with CCA, the overall survival for these patients remains dismal. Utilizing combination approaches with targeted therapies may offer some patients more benefit than single agents [137,138,139]. Mechanisms of resistance to single agent therapies may include RNA silencing [140] as well as multiple gene driven pathogenesis, which could be overcome with ‘multiomic’ patient diagnostics using genomic, proteomic, transcriptomic, and immunomic data as well as n-of-1 customized combination therapeutic approaches [141,142,143,144]. Further, using patient-specific immunomic data may allow for a tailored immunotherapy-targeted treatment as well as being informed by specific genomic aberrations and the most logical immunotherapeutic target [145]. A next step in improving outcomes in these patients likely involves identifying more therapeutic targets as well as overcoming secondary resistance mechanisms via targeting as many genomic alterations present within a specific tumor as possible [144,146,147].

## Figures and Tables

**Table 1:** The frequency of genetic alterations according to the anatomic location of CCA.

CCA Subtype	iCCA (Affects Bile Ducts within the Liver)	eCCA (Affects Bile Ducts Outside of the Liver)
ARID1A	18–23% [23,24]	14% [25]
BAP1	15–20% [23,24]	--
BRAF V600E	1.5% [26]	--
BRCA1	0.4% [27]	2% [27]
BRCA2	2.8% [27]	2.5% [27]
CDH1	11.8% [28]	--
CDKN2A/B	9–27% [23,24]	9–28% [25,29,30]
ERBB2/HER2	5.8% [31,32]	1.3–20% [25,31,32]
FGFR2 fusion	10–16% [23,33]	0 [23,33]
IDH1/2	13–30% [23,34]	4.7% [25]
KRAS	7–54% [23,24,25]	36.7–46% [25,29,30]
MSI-H/dMMR	4.7–18.2% [35]	4% [25]
PI3K	7% [23,25]	5% [23,25]
SMAD4	--	10.7% [25]
TP53	18–27% [23,24]	18–68% [25,29,30]

Abbreviations: ARID1A: AT-rich interaction domain 1A; BAP1: BRCA1-associated protein 1; BRCA: breast cancer antigen; CCA, cholangiocarcinoma; CDH1: cadherin-1; eCCA: extrahepatic CCA; ERBB: erythroblastic leukemia viral oncogene homolog; FGFR: fibroblast growth factor receptor; iCCA: intrahepatic CCA; IDH: isocitrate dehydrogenase; KRAS: Kirsten rat sarxoma viral oncogene homolog; MSH, microsatellite instability; MSI: molecular microsatellite instability; PIK3: phosphoinositide-3-kinase; SMAD4: mothers against decapentaplegic homolog 4; TMB, tumor mutational burden.; TP53: tumor protein 53.

**Table 2:** Examples of targeted therapy trials.

Target (Gene)	% in CCA	FDA-Approved Drug	Date of Approval	Trials	Total	ORR	OS	PFS	Disease-Free Survival	Duration of Response	Major Adverse Events (Grade ≥ 3)
					No. of CCA/ BTCs (%)						
*NTRK* gene fusion-positive	3–9% [46]	Entrectinib	15 August 2019	Drilon et al., 2017 [47]	55 (100%)	100% (95% CI: 44 to 100)	NR	NR	NR	For the three patients, the DoR was 2.6 months, 4.6 months, and 15.1 months	Fatigue/asthenia: 5 (4%) Weight increase: 2 (2%) Diarrhea: 1 (1%) Arthralgia: 1 (1%)
					NR	NR	NR	NR	NR	NR	
				Doebele et al., 2020 [48]	54 (100%)	57% (95% CI: 43.2 to 70.8)	21 months (95% CI 14.9 to NE)	11.2 months (95% CI 8.0 to 14.9)	NR	10.4 months (95% CI: 7.1 to NE)	Anemia: 8 (12%) Increased weight: 7 (10%) Fatigue: 5 (7%)
					1 (2%)	NR	NR	NR	NR	NR	
		Larotrectinib	26 November 2018	Drilon et al., 2018 [49]	55 (100%)	80% (95% CI: 67 to 90)	NR	Not reached	NR	Not reached	Anemia: 114 (11%) Increased weight: 73 (7%) Decreased neutrophil count: 73 (7%) Increased ALT and AST: 73 (7%)
					2 (4%)	50% objective tumor shrinkage	NR	NR	NR	NR	
*BRAF-V600E*	1.5 [26]	Trametinib plus dabrafenib	22 June 2022	Subbiah et al., 2020 [50]	43 (100%) *	51% (95% CI: 36 to 67)	14 months (95% CI: 10 to 33)	9 months (95% CI: 5 to 10)	NR	9 months (95% CI: 6 to 14)	γ-glutamyltransferase increased: 5 (12%) Decreased WBC count: 3 (7%) Pyrexia: 3 (7%)
				Salama et al., 2020 [51]	29 (100%)	38% (95% CI: 22.9% to 54.9%)	28.6 months	11.4 months (90% CI: 8.4 to 16.3)	NR	25.1 months (90% CI: 12.8 to NE)	Fatigue: 4 (11.4%) Decreased neutrophil count: 3 (8.6%) Decreased WBC count: 3 (8.6%)
					4 (13.8%)	75% (3/4 pts), one is ongoing for 29 months	NR	NR	NR	NR	
*FGFR2* fusion or rearrangements	10–16% [33]	Pemigatinib	17 April 2020	Abou-Alfa et al., 2020 [52]	107 (100%) *	35.5% (95% CI: 26.5 to 45.4%)	21.1 months (95% CI: 14.8 to NE)	6.93 months (95% CI: 6.18 to 9.59)	NR	7.5 months (95% CI: 5.7 to 14.5)	Hypophosphataemia: 10 (7%) Stomatitis: 8 (5%) Arthralgia: 6 (4%) Palmar-plantar erythrodysesthesia: 6 (4%)
		Infigratinib	28 May 2021	Javle et al., 2021 [53]	108 (100%) *	23.1 (95% CI 15.6 to 32.3%)	12.5 (95% CI: 9.9 to 16.6)	6.8 (95% CI: 5.3 to 7.6)	NR	5.4 (95% CI: 3.7 to 7.4)	Hypophosphatemia: 10 (14.1%) Hyperphosphatemia: 9 (12.7%) Hyponatremia: 8 (11.3%)
		Futibatinib	30 September 2022	Goyal et al. [54]	103 (100%) *	41.7%	20.0	8.9	NR	9.5	NR
MSI-H/dMMR tumors	4.7–18.2% [35]	Pembrolizumab	16 June 2020	Le et al., 2017 [55]	86 (100%)	53% (95% CI: 42% to 64%)	Not reached 2-year OS: 64% (95% CI: 53% to 78%)	Not reached 2-year PFS: 53% (95% CI: 42% to 68%)	NR	NR	Diarrhea/colitis: 5 (6%) Pancreatitis/Hyperamylasemia: 5 (6%) Fatigue: 2 (2%) Anemia: 2 (2%)
					4 (4.7%)	NR	NR	NR	NR	NR	
				Marabelle et al., 2019 [56]	233 (100%)	34.3% (95% CI: 28.3 to 40.8)	23.5 months (95% CI: 13.5 to NR)	4.1 months (95% CI: 2.4 to 4.9)	NR	Not reached (range, 2.9 to 31.3+ months)	Fatigue: 2 (0.9%) Asthenia: 1 (0.4%)
					22 (9.4%)	40.9% (95% CI: 20.7 to 63.6) in CCA pts	24.3 months (95% CI: 6.5 to NE) in CCA pts	4.2 months (2.1 to NE) in CCA pts	NR	NR	
		Dostarlimab	17 August 2021	Andre et al., 2021 (Abstract) [57]	106 (100%)	38.7% (95% CI: 29.4 to 48.6)	NR	NR	NR	Not reached	Lipase increased: 2 (1.4%)
					2 (1.9%)	100% CR	NR	NR	NR	NR	
*IDH1*	13% [34]	Ivosidenib	25 August 2021	Lowery et al., 2019 [58]	73 (100%) *	5% (95% CI: 1.5 to 13.4)	13.8 months (95% CI: 11.1 to 29.3)	3.8 months (95% CI: 3.6 to 7.3)	NR	NR	Ascites: 4 (5%) Anemia: 3 (4%) Fatigue: 2 (3%)
				Abou-Alfa et al., 2020 [34]	185 (100%) *	2% (95% CI: 0.5 to 6.9)	10.8 months (95% CI: 7.7 to 17.6)	2.7 months (95% CI: 1.6 to 4.2)	NR	NR	Ascites: 9 (7%) Aspartate aminotransferase increased: 6 (5%) Anemia: 4 (3%) Fatigue: 4 (3%)
HER2-positive tumor	5.8% of iCCA and 13–20% of eCCA [31,32]	Pertuzumab plus trastuzumab	Not yet approved in CCA	Javle et al., 2021 [59]	39 (100%) *	23% (95% CI: 11 to 39)	10.9 months (95% CI: 5.2 to 15.6)	4.0 months (95% CI: 1.8 to 5.7)	NR	10.8 months (95% CI: 0.7 to 25.4)	Increased alanine aminotransferase: 5 (13%) Increased aspartate aminotransferase: 5 (13%) Blood alkaline phosphatase increased: 4 (10%)
*RET* fusion-positive	NR	Pralsetinib	Not yet approved in CCA	Subbiah et al., 2022 [60]	23 (100%)	57% (95% CI: 35%–77%)	13.6 months (95% CI: 7.5 to NE)	7.4 months (95% CI: 5.1 to 13.6)	NR	11.7 months (95% CI: 5.5 to 19.0)	Neutropenia: 9 (31%) Anemia: 4 (14%) Increased AST: 3 (10%)
					3 (13%)	66.7%	NR	NR	NR	NR	
		Selpercatinib	21 September 2022	Subbiah et al., 2022 [61]	45 (41 evaluated for efficacy)	43.9% (95% CI 28.5–60.3)	18.0 months (95% CI: 10.7– Not estimated) ***	13.2 months (95% CI: 7.4–26.2)	NR	24.5 (95% CI: 9.2–Not estimated)	Hypertension (22%) Increased alanine aminotransferase (16%) Increased aspartate aminotransferase (13%).
					2 (1 evaluated for efficacy)	100%				5.6 months	

* All patients were CCA/BTCs; *** investigator assessed. Abbreviations: BTCs: biliary tract cancers; CCA: cholangiocarcinoma; CI: confidence interval; DoR: duration of response; eCCA: extrahepatic cholangiocarcinoma; iCCA; intrahepatic cholangiocarcinoma; NE: not estimated; NR: not reported; OS: overall survival; ORR: objective response rate; PFS: progression-free survival; WBCs: white blood cells.

**Table 3:** Examples of ongoing trials in CCA.

Target	Phase	Clinical Trial Identifier	Treated Cancer Group	Experimental Arm	Control Arm	Primary Outcome	Secondary Outcome (Main)
First Line							
*FGFR2* fusion/rearrangement	III	NCT03656536	CCA	Pemigatinib	Gemcitabine/Cisplatin	PFS	OS, OR, DOR, DCR
	III	NCT03773302	CCA	Infigratinib	Gemcitabine/Cisplatin	PFS	OS, DCR, DOR, BOR
	III	NCT04093362	iCCA	Futibatinib	Gemcitabine/Cisplatin	PFS	OS, safety, ORR, DCR
	II	NCT03230318	iCCA	Derazantinib	None	ORR, PFS	OS, safety, DCR
	I/II	NCT04526106	iCCA and other advanced tumors	RLY-4008	None	ORR, MTD, safety	DOR, DCR, pharmacokinetics
HER 2 mutations	II	NCT03613168	BTCs	Trastuzumab plus gemcitabine/cisplatin	None	BOR, safety	PFS, OS
	I/II	NCT02992340	BTCs	Varlitinib plus gemcitabine/cisplatin	None	MTD, safety, PFS, ORR	OS, DOR, DCR, PK
Subsequent lines							
*NTRK* gene fusion-	II	NCT04879121	Advanced solid tumors	Larotrectinib	None	ORR	PFS, OS, safety, DOR, GMI, CBR
	II	NCT03213704	Advanced solid tumors	Larotrectinib	None	ORR	PFS, safety, PK, changes in tumor genomics
Non-V600E BRAF mutations	II	NCT03839342	Advanced solid tumors	Bimimetinib + Encorafenib	None	ORR	PFS, safety, DCR
	I	NCT04190628	Advanced solid tumors	ABM-1310	None	MTD	PFS, OS, safety, PK, ORR, DCR, DOR
	I	NCT04249843	Advanced solid tumors	BGB-3245	None	Safety, MTD	PFS, OS, PK, ORR, DCR, DORƒ
	I	NCT04418167	Advanced solid tumors	JSI-1187 monotherapy or in combination with dabrafenib	None	Safety	PFS, OS, ORR, DOR, time to response, DCR, PK
*IDH1/2* mutations	II	NCT02428855	iCCA	Dasatinib	None	ORR	PFS, OS, safety
	II	NCT03212274	CCA	Olaparib	None	ORR	PFS, OS, safety
	II	NCT03878095	CCA	Ceralasertib + Olaparib	None	ORR	PFS, OS, safety, DOR
	I/II	NCT02273739	Advanced solid tumors	Enasidenib	None	DLT, ECOG	Plasma concentration metrics
	I	NCT04521686	CCA	LY3410738 LY3410738 + Gemcitabine/Cisplatin		MTD	ORR, safety and tolerability, efficacy, PK
dMMR/MSI-H	I/II	NCT04800627	Advanced solid tumors	Pevonedistat in combination with Pembrolizumab	None	Recommended phase 2 dose, ORR	PFS, OS, safety, changes in protein misfolding
HER 2 mutations	II/III	NCT03093870	BTCs	Varlitinib with Capecitabine	Capecitabine	ORR, PFS	OS, safety, DOR, DCR, tumor size, ECOG
	II	NCT03185988	Metastatic carcinoma of digestive system including BTCs	Trastuzumab plus 5-FU or IRI or Capecitabine	None	RR	OS, PFS, DCR, DOR, time of response, ECOG
	II	jRCT2031180150	Advanced solid tumors	Trastuzumab and Pertuzumab	None	ORR	PFS, OS, safety, DOR
	II	NCT02999672	CCA	Trastuzumab emtansine	None	BOR	PFS, OS, safety, PK
	II	NCT02675829	Advanced solid tumors	Ado-Trastuzumab emtansine	None	ORR	None
	II	NCT04482309	Advanced solid tumors	Trastuzumab Deruxtecan	None	ORR	OS, PFS, safety, DOR, DCR, PK, immunogenicity
	I/II	NCT03410927	Advanced solid tumors	TAS0728	None	Safety, ORR	OS, DOR, PK, DCR
	I	NCT04764084	CCA	Niraparib + Anlotinib	None	DLT, MTD	PFS, ORR
	I	NCT02892123	Advanced solid tumors	Zanidatamab plus chemotherapy	None	MTD, Safety	PFS, ORR, PK, antidrug antibodies
	I	NCT02564900	Non-breast/non-gastric solid tumors	Trastuzumab Deruxtecan	None	ORR	DCR, BOR, DOR, PFS, OS, pharmacokinetics, safety
BAP1 and other DDR genes	II	NCT03207347	CCA	Niraparib	None	ORR	PFS, OS, safety
DNA repair gene mutation	II	NCT03207347	CCA	Niraparib	None	ORR	PFS, OS, safety
Matched molecular therapy							
Matched molecular therapy	N/A	NCT04504604	Rare tumors	FoundationOne CDx and FoundationOne Liquid CDx	None	% who receive a molecularly targeted matched, PFS	Tumor molecular profiles correlation to treatment outcome.

Abbreviations: BOR: best overall response; BTCs: biliary tract cancers; CBR: clinical benefit rate; CCA: cholangiocarcinoma; DCR: disease control rate; DLT: dose-limiting toxicity; DOR: duration of response; ECOG: Eastern Cooperative Oncology Group; GMI: growth modulation index; iCCA: intrahepatic cholangiocarcinoma; MTD: maximum tolerated dose; N/A: not applicable; ORR: overall response rate; OS: overall survival; PFS: progression-free survival; PK: pharmacokinetics.
